# Association between the Interaction of Key Genes Involved in Effector T-Cell Pathways and Susceptibility to Developallergic Rhinitis: A Population-Based Case-Control Association Study

**DOI:** 10.1371/journal.pone.0131248

**Published:** 2015-07-21

**Authors:** Yuan Zhang, Jingyun Li, Chengshuo Wang, Luo Zhang

**Affiliations:** 1 Department of Otolaryngology Head and Neck Surgery, Beijing TongRen Hospital, Capital Medical University, Beijing 100730, PR China; 2 Beijing Key Laboratory of nasal diseases, Beijing Institute of Otolaryngology, Beijing 100005, PR China; 3 Department of Allergy, Beijing TongRen Hospital, Capital Medical University, Beijing 100730, PR China; Academic Medical Centre/University of Amsterdam, NETHERLANDS

## Abstract

**Background:**

Evidence suggests that interaction between key genes mediating signaling and transcriptional networks involving effector T-cell responses may influence an individual’s susceptibility to develop allergic rhinitis(AR).

**Objective:**

The aim of this study was todetermine whether specific interactions between key genes involved in effector T-cell pathways are associated with an individual’s susceptibility to develop AR in Han Chinese subjects.

**Method:**

A cohort of 489 patients with AR and 421 healthy controls was enrolled from the Han Chinese population in Beijing, China. AR was established by questionnaire and clinical examination, and peripheral blood was drawn from all subjects for DNA extraction. A total of 96 single nucleotide polymorphisms (SNPs) in 26 reprehensive candidate genes involved in T helper 1 (Th1), Th2, Th17, Th9 and T regulatory cell pathways were selected from the International Haplotype Mappingdatabase for Han Chinese in Beijing (CHB) population, and IlluminaGoldenGate assay was conducted for SNP genotyping. The PLINK software package was used to perform statistical analyses.

**Results:**

Simple SNP-phenotype association analysis using logistic regression showed SNP rs8193036 in IL17A gene, rs2569254 in IL-12 and rs1898413 in RORα weresignificantlyassociatedwith AR.Simple SNP-phenotype association analysis with genetic models demonstrated thatrs2569254 in IL-12, rs1031508 in STAT4, and rs3741809 in IL-26 were likely to be recessive, rs8193036 in IL17A allelic, rs897200in STAT4 genotypic, and rs1898413 in RORα dominant. Epistasis analyses exhibited that 83 SNPs in 23 genes were significantly interactive; of which 59 interactions/SNP pairs demonstrated OR values higher than 2 or lower than 0.5, and 12 interactions/SNP pairs OR values higher than 4 or lower than 0.25. STAT3, RORα and IL-26, involved in Th17 pathway,were the mostfrequentlyinteractive genes.

**Conclusion:**

This study suggests that interactions between several SNPs in key genes involved in effector T-cell pathways are likely to influence an individual’s susceptibility to develop AR.

## Introduction

Allergic rhinitis (AR) is a serious systemic disease which, with comorbid asthma, causes major illness and disability worldwide. AR has increased in prevalence over the last decade and currently affects up to 40% of the population worldwide[1]. Recent data from mainland China indicates that the prevalence of self-reported AR in major cities across China is high and ranges between 8.7%-24.1%[2]; whichis in accordance with the trends noted for AR prevalence in other developing countries[3].

Allergic sensitization; defined by production of IgE against environmental antigens such as house dust mite and grass pollen; can lead to diseases that include AR, asthma and atopic dermatitis[4]. The immunological mechanisms underlying allergic sensitization involve functional T cell subsets including T helper 1 (Th1) and T helper 2 (Th2) cells that display polarized cytokine profiles; with a general consensus among researchers that a weak Th1 imprinting and an unrestrained Th2 response allows an increase in allergic responses[5]. Increasing evidence from studies investigating the mechanisms underlying the pathogenesis of allergic diseases has further implicated the important contributions of T regulatory (Treg) cells[6]and the newly described proinflammatoryTh17 cell lineage[7] in this process. It has been demonstrated that the commitment of peripheral T-cell clones to undergo differentiation into one of these lineages is shaped by self-reinforcing transcriptional circuitries that center on key transcriptional regulators: T-box expressed in T cells (Th1), GATA-3 (Th2), forkhead box p3 (Treg), and retinoid-related orphan receptor gt/retinoid-related orphan receptor a (Th17)[8]. Moreover, Th9 cells; a novel, distinct population of effector Th cells; has been shown to be involved in tissue inflammation[9],whereas IL-9 in combination with TGF-β has been reported to contribute to Th17 cell differentiation[10].

It is believed that allergic diseases, including AR, are complex genetic diseases resulting from the effect of both multiple genetic and interacting environmental factors and that gene-gene and gene-environment interactions may contribute to the complexity of the diseases[11]. Gene-gene interactions; including dominant, recessive effects as well as epistasis; are defined as the functional interplay between genetic variants within a pathway. Furthermore, it has been suggested that each variant typically has modest effects in isolation, but synergizes effectively with other variants to magnify the impact on disease risk[12].

In view of this evidence, we hypothesized that the key genes mediating signaling and transcriptional networks involving effector T-cell responses are likely to be strong candidate genes, which may influence an individual’s risk to develop AR. Furthermore, there may exist gene-gene interactions among these candidate genes within a single pathway or between different pathways. The aim of this study was therefore to examine whether specific interactions between key candidate genes involved in effector T-cell pathways are associated with an individual’s susceptibility to develop AR in Han Chinese subjects.

## Materials and Methods

A population-based case-control association study design was used to assess susceptibility to AR, conferred by SNPs in effector T-cell pathways gene regions.

### Study subjects

Four hundred and eighty-nine consecutive adult subjects suffering from AR were recruited from the outpatient clinic of Allergic rhinitis center at Beijing TongRen Hospital, during the study period from February 2010 to February 2011.

All subjects had a history of AR for at least 1 year and fulfilled all AR criteria of the Allergic Rhinitis and its Impact on Asthma (ARIA) guidelines [13], including i) presence of persistent or discontinuous symptoms of anterior rhinorrhoea, continuous sneezing, nasal obstruction and itching, ii) demonstration of a pale and edematous nasal mucosa, nasal discharge and swollen inferior turbinates by nasal endoscopy, and iii) positive skin prick test (SPT) to a panel of common allergens as shown below (Allergopharma, Reinbeck, Germany) and positive serum antigen-specific IgE, measured by the ImmunoCAP 100 system (Pharmacia, Uppsala, Sweden). A diagnosis of AR was further confirmed by the presence of symptoms induced by exposure to an allergen shown to produce a strong positive skin test response.

The tested antigens included house dust mite (HDM) (Der f and Der p); seasonal grass pollens (Giant Ragweed; Mugwort; Lamb’s quarers; Humulus; Chenopodium album); animal hair (dog and cat); molds (indoor and outdoor mustiness or floricultural environment) and cockroach. A positive SPT result was defined as a wheal greater than or equal to one half of the diameter of the histamine control and at least 3 mm larger than the diameter of the negative control[14]. Subjects were also considered to be sensitized to allergens when the serum IgEwas≥0.35 kU/l.

AR subjects with i) co-morbid asthma, eczema, or any other allergic disease; ii) hypertension, diabetes or other chronic diseases; or iii) tumor in the nasal cavity or any other inflammatory nasal disease were excluded. The diagnosis of asthma was confirmed by a chest physician according to Global Initiative for Asthma (GINA) guidelines[15].

A total of 421 adult healthy control volunteers were also recruited during the study period from an ethnically similar local population to determine background population allele frequencies. None of the control subjects had a history of allergic or any nasal disease, nor demonstrated any abnormal clinical features in the nasal cavity or a positive SPT to any of the common allergens as shown above.

All subjects were of Han Chinese ethnic origin from the Beijing region, China, and provided written informed consent prior to entry in the study. The study protocol was approved by the Ethics Committee of Beijing TongRen Hospital and performed in accordance with the guidelines of the World Medical Association's Declaration of Helsinki.

### Selection of polymorphisms in the key genes involved in effector T-cell pathways

The International Haplotype Mapping (HapMap) (www.hapmap.org) SNP databases were used to select tag SNPs (tSNPs) in the candidate genes regions; with the screened region extended 10 kilobases upstream of the annotated transcription start site and downstream at the end of the last exon in the gene. The tSNPs were selected to extract most genetic information in the region using the Han Chinese in Beijing (CHB) population genotyping data from the HapMap database (HapMap data rel 27 Phase II+III, Feb2009)[16]. Genotyping data were obtained for 138tSNPs for key genes involved in effector T-cell pathways in the dataset and loaded in the Haploview software version 4.2 (http://www.broad.mit.edu/haploview/haploview-downloads)[17]. Further selection of the eventual tSNPs to be investigated was then made using a pair-wise tagging algorithm[17]; setting the Hardy-Weinberg p value, minor allele frequency (MAF), and r^2^ threshold values at 0.01, 0.05 and 0.8, respectively. The linkage disequilibrium (LD) pattern of the candidate genes in the CHB population exhibited strong LD in several groups of tSNPs (r^2^ greater than or equal to 0.8), indicating that most common SNPs could be captured by a subset of tSNPs[18]. Consequently, 96 SNPs were selected to represent the entire 26 genes for genotyping, as shown in Table 1.

**Table 1:** Candidate SNPsin the key genes involved in effector T-cell pathways.

Pathway	Gene	SNP
Treg	IL-2	rs2069772
	IL-10	rs3024495; rs3024490; rs3021094; rs3790622; rs1800893
	FOXP3	rs2232365;rs3761548;rs3761549
	STAT6	rs324015; rs167769
Th1	IL-12	rs3212219; rs11574790; rs2569253; rs2569254; rs1433048
	IFN-γ	rs1131964; rs11701402; rs8128785; rs2073362
	T-bet,	rs17244587; rs11657388
	STAT1	rs34997637; rs7575823; rs2280235; rs2030171; rs3771300; rs2066804; rs1467199
	STAT4	rs1551440; rs11889341; rs6715106; rs3024889; rs13017460; rs1031508; rs7566274; rs897200
Th2	IL-4	rs2243240; rs2243248; rs2243263; rs2243283
	IL-25	rs10137082; rs3811178; rs10135798
	IL-5	rs2069812
	IL-13	rs1295687; rs2069744; rs1881457
	IL-31	rs7977932
	IL-33	rs1317230; rs1332290
	GATA3	rs444929; rs369421; rs10752126; rs406103
Th17	IL-6	rs13306433; rs1800796
	IL-21	rs2069762
	IL17A	rs3819025; rs8193039; rs3748067; rs2275913; rs4711998; rs8193036; rs3819024
	IL-22	rs2046068; rs2227501; rs17224704; rs1179251; rs1182844; rs2227481
	IL-26	rs4913419; rs11177102; rs3741809; rs10784693; rs3782555; rs3814240
	STAT3	rs3816769; rs1053005; rs17405722
	RORα	rs2162069; rs1898413; rs12594972; rs17237290; rs12905435; rs2289163; rs11635975
Th9	IL-9	rs2069868; rs2069870; rs31564
	NF-κB	rs3774932; rs1598861; rs230541; rs4648037; rs4648110
	GATA1	rs5906709

SNP, single nucleotide polymorphis

### Single nucleotide polymorphism genotyping

DNA was isolated from peripheral blood leukocytes using the DNA Isolation Kit for Mammalian Blood (Roche, Indianapolis, USA), and stored at 4°C prior to further investigation within 2 days. GoldenGate assay (IlluminaInc, San Diego, USA); capable of multiplexing from 96 to 1,536 SNPs in a single reaction over a 3-day period; was used for SNP genotypingaccording to the manufacturer’s instructions. A 96-SNP GoldenGate assay was designed using SNPs selected from the 26 genes of the T-cell pathways. To ensure the accuracy of the genotyping, quality control was performed using exclusion criteria for SNPs as follows: 1) maximum per-person missing rate>5%; 2) Hardy-Weinberg disequilibrium p-value< 0.001; 3) maximum per-SNP missing rate > 5%; 4) minor allele frequency< 0.01.

### Statistical analyses

Simple SNP-phenotype associations were performed using logistic regression, as well as a series of genetic models (i.e. full model) including epistasis, allelic, genotypic, additive, dominant, recessive, and trend models, which were derived and analyzed using the PLINK software[19], a free, open-source whole genome association analysis toolset, designed to perform a range of basic, large-scale analyses in a computationally efficient manner. Epistasis model was defined as Y ~ b0 + b1.A + b2.B + b3.AB + e, in which A and B represent allele dosage of each SNP and AB represents the interaction. The test for interaction was based on the coefficient b3. *P*-value<0.05 was considered to be significant.

## Results

### Population characteristics

The demographic characteristics of the study population are shown in Table 2. Both the AR and control groups were well matched with respect to age and gender. The mean ages of the AR and control groups were 34 and 37 years old, respectively and both groups consisted of more males than females (AR group = 55.6%/ 44.4% males/females; control group = 52.0%/ 48.0% males/females). The differences in neither the age (*P* = 0.063)nor the ratios for males/females(*P* = 0.277) between the control and AR groups were not significantly different. The mean total serum IgE measurements for AR and control groups were significantly different (315.8 ± 489.6 and 73.4 ± 124.3IU/ml respectively; *P* = 0.0000). Moreover, 353 (72.2%), 46 (9.4%), and 90 (18.4%) of AR subjects, respectively, were found to be allergic to house dust mites, pollens, and other allergens. Overall, the 72.2%of HDM-sensitized subjects were polysensitized and the remaining 27.8% subjects were monosensitized to seasonal grass pollens, animal hair, moldsor cockroach.

**Table 2:** Demographic and clinical characteristics of the study groups.

Demographic Index	Allergic rhinitis (n = 489)	Control (n = 421)
Age: Mean (Range) (years)	34.1 ± 12.7 (18–70)	36.9 ± 14.3 (18–80)
Sex: Male/Female, No. (%)	272 (55.6) /217 (44.4)	219 (52.0) / 202 (48.0)
Total IgE: Mean (Range), kU/l	315.8 ± 489.6 (1.06–4552)	73.4 ± 124.3 (2–978)
Allergen category: No. (%)		
House dust mites	353 (72.2)	-
Pollens	46 (9.4)	-
Other allergens	90 (18.4)	-

### SNP genotyping

The overall mean genotyping success rate for the loci was 99.3%; however, 32 individuals including 11 AR cases and 21 controls were removed from the analysis for low genotyping (maximum per-person missing rate >5%). Two SNPs were removed for failing the Hardy-Weinberg equilibrium test (*P*< 0.001). Two SNPs failed missingness test (maximum per-SNP missing rate >5%) and three SNPs failed frequency test (minor allele frequency<0.01), and uniquely three SNPs were removed. After quality control, 91 SNPs and 878 individuals (478 cases and 400 controls)were evaluable; providing a total genotyping rate of 99.4% for the remaining SNPs.

### Individual SNP association analysis

Significant simple SNP-phenotype association results with logistic regression model are shown in Table 3. The SNP rs8193036 in IL17Agene was the most significant with *P* = 0.019, and OR value of 0.78, indicating that the allele A was likely to play a protective role. Similarly, the SNPS rs2569254 in IL-12 and rs1898413 in RORα demonstrated P-values of around 0.04; with the OR value of 0.78 for the allele A of rs2569254 indicating that this was likely to be protective, and OR value of 1.30for the allele A of rs1898413 indicating that this was likely to be aggressive.

**Table 3:** Simple SNP-phenotype association results with logistic regression.

Gene	Chromosome	SNP	Alleles	OR	95%CI	STAT	*P-value*
IL-12	5	rs2569254	A/G	0.78	0.62–0.99	-2.058	0.040
IL17A	6	rs8193036	A/G	0.78	0.64–0.96	-2.349	0.019
RORα	15	rs1898413	A/G	1.30	1.00–1.69	2.014	0.044

SNP, single nucleotide polymorphism; OR, odds ratio; STAT, Chi-square statistic

Simple SNP-phenotype association results with genetic models are shown in Table 4. Under the allelic model, rs8193036 in IL17A, rs2569254 in IL-12 and rs1898413 in RORα were found to be significant (*P* = 0.018, 0.040, 0.040, respectively), whereas under the genotypic model, rs897200 in STAT4, rs2569254 in IL-12, andrs1031508 in STAT4 were significant (*P* = 0.026, 0.037, 0.046, respectively). Similarly, under the dominant model, rs8193036 in IL17A, rs897200 in STAT4, and rs1898413 in RORα were significant (*P* = 0.026, 0.036, 0.039, respectively) while under the recessive model, rs2569254 in IL-12, rs1031508 in STAT4, and rs3741809 in IL-26 were significant (*P* = 0.015, 0.024, 0.033, respectively). Under the trend model, rs8193036 in IL17A, rs2569254 in IL-12, and rs1898413 in RORα were significant (*P* = 0.019, 0.039, 0.043, respectively). Uniquely, six SNPs were significant; of which rs2569254 in IL-12, rs1031508 in STAT4, and rs3741809 in IL-26were likely to be recessive (*P* = 0.015, 0.024, 0.033, respectively), rs8193036 in IL17A was likely to be allelic (*P* = 0.018), rs897200 in STAT4 was likely to be genotypic (*P* = 0.026), and rs1898413 in RORα was likely to be dominant (*P* = 0.039).

**Table 4:** Simple SNP-phenotype association results with full model.

SNP	Chr	Position	Gene	Model	Alleles	Case	Control	*P-value*
rs1031508	2	191145485	STAT4	GENO	A/G	62/196/219	33/187/180	0.046
rs1031508	2	191145485	STAT4	REC	A/G	62/415	33/367	0.024
rs897200	2	191153045	STAT4	GENO	G/A	116/215/146	87/216/97	0.026
rs897200	2	191153045	STAT4	DOM	G/A	331/146	303/97	0.036
rs2569254	5	159324241	IL-12	GENO	A/G	12/154/311	23/135/242	0.037
rs2569254	5	159324241	IL-12	TREND	A/G	178/776	181/619	0.039
rs2569254	5	159324241	IL-12	ALLELIC	A/G	178/776	181/619	0.040
rs2569254	5	159324241	IL-12	REC	A/G	12/465	23/377	0.015
rs8193036	6	52185695	IL17A	TREND	A/G	270/686	267/531	0.019
rs8193036	6	52185695	IL17A	ALLELIC	A/G	270/686	267/531	0.018
rs8193036	6	52185695	IL17A	DOM	A/G	231/247	223/176	0.026
rs3741809	12	68201364	IL-26	REC	G/A	105/366	65/329	0.033
rs1898413	15	60528610	RORα	TREND	A/G	167/789	111/689	0.043
rs1898413	15	60528610	RORα	ALLELIC	A/G	167/789	111/689	0.040
rs1898413	15	60528610	RORα	DOM	A/G	151/327	101/299	0.039

Note: SNP, single nucleotide polymorphisms; Chr, chromosome; ALLELIC, allelic model; GENO, genotypic model; DOM, dominant model; REC, recessive model; TREND, trend model.

### Epistasis analysis

The data for epistasis analyses with *P* values< 0.05 are shown in S1 Table. Overall 83 of the 91 (91.2%) SNPs in 23 of the 26 (88.5%) genes were detected to be significantly interactive; of which 59 interactions/SNP pairs demonstrated OR values higher than 2 or lower than 0.5, and 12 interactions/SNP pairs OR values higher than 4 or lower than 0.25.

**Fig 1:**
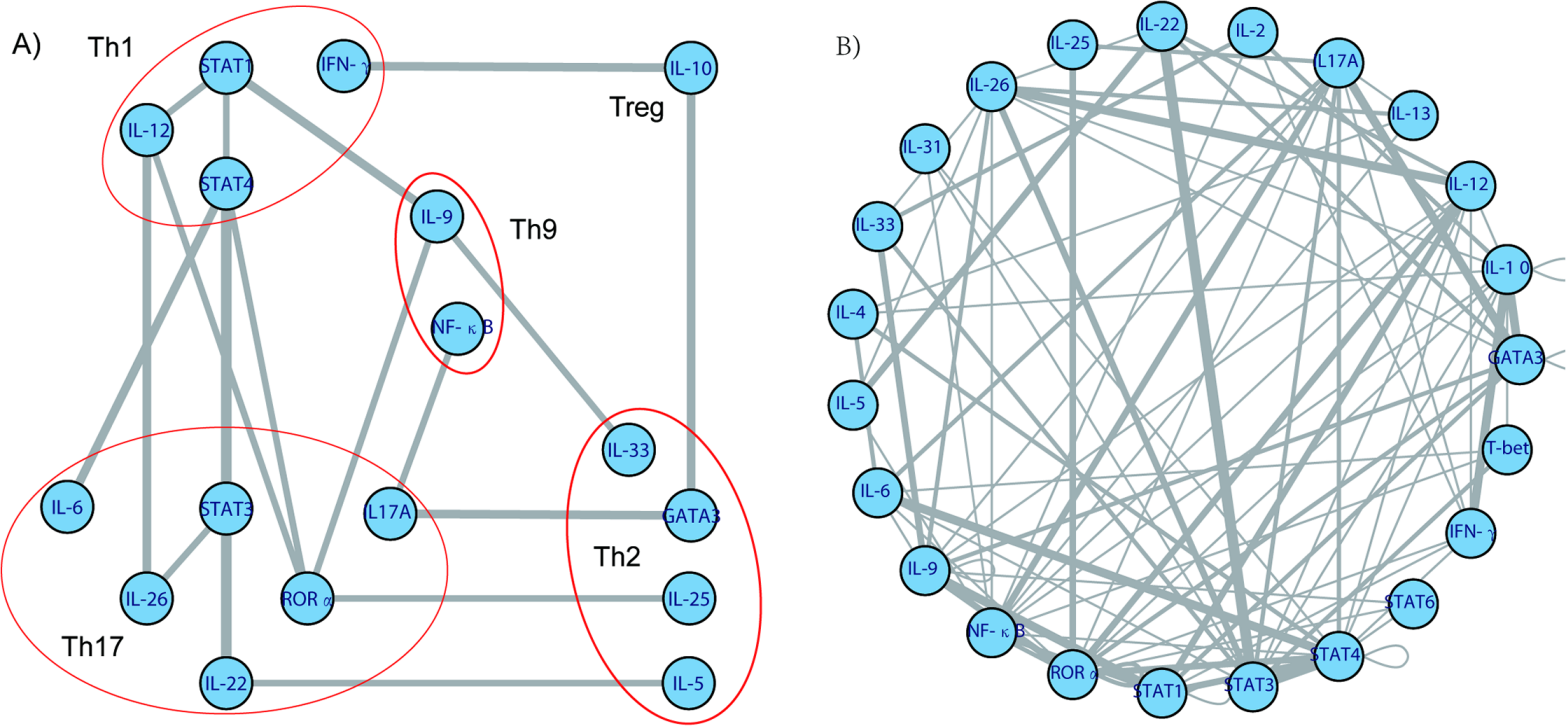
Networkof the interactive genes. Figure A shows the frameworkof the interactivepatternamong the Treg, Th1, Th2, Th9 and Th17 pathways. Figure B shows all the detailed relationships among the Treg, Th1, Th2, Th9 and Th17 pathways.

Fig 1 shows the frameworkof the interactivepatternamong the Treg, Th1, Th2, Th9 and Th17 pathways-associated genes, based on data shown in S1 Table. When any two genes had more than two significant interactive SNP pairs, they were regarded to be mainly interactive with each other. Using this definition, STAT4 was mainly interactive with STAT1, STAT3, RORα and IL-6; STAT1 mainly interactive with IL-9 and IL-12; STAT3 mainly interactive with IL-22 and IL-26; RORα mainly interactive with IL-9, IL-12 and IL-25; IL-9 mainly interactive with IL-33; and IL-22 mainly interactive with IL-5. These 12 genes mainly combined the Th1 and Th17 pathways (Fig 1A), to form one sub-network, which was extended to a slightly bigger sub-network by inclusion of IFN-r, IL-10, GATA3, IL17A and NF-κB genes, to encompass all five effector T-cell pathways (Fig 1A).

Relaxation of the definition for interactive genes, from “more than two interactive SNP pairs” to “not less than two interactive SNP pairs”, indicated that another four genes; including IL-2 (Treg), T-bet (Th1), IL-4 and IL-13 (Th2); were also interactive with other genes in these T cell pathways (Fig 1B).

## Discussion

In this study we aimed to explore the contribution of genetic variations and interactions between key genes involved in effector T-cell pathways; includingTh1, Th2, Treg, Th17 and Th9;in susceptibility towards AR, in a cohort of Han Chinese subjects. In the present study, the majority (72.2%) of the subjects was polysensitized and the sesubjects were allergic to house dust mites (HDMs) allergens Der f and Der p, which have been shown to be the most common sensitizing inhalant allergens in Beijing and many parts of China [20]. Although this group of polysensitized subjects is representative of the general AR population in Beijing, it is possible that stratification analysis for the presence of different allergens may influence the association status of the genetic variants [21,22].Our study has nevertheless demonstrated that there were 83significant interacting SNPs in 23 key genes-associated with the mediation of these T-cell pathways. Simple SNP-phenotype association analysis of AR risk, indicated that allele A of SNP rs8193036 in the IL17A gene and allele A of rs2569254 in the IL-12 gene were likely to exhibit a significantly protective role against the development of AR, whereas allele A of rs1898413 located in RORα region waslikelytoexhibit anaggressive effect. Similarly, simple SNP-phenotype association analysis employing different genetic models revealed significant associations within several SNP loci in selected candidate genes. Furthermore, epistasis analyses demonstrated relatively clear interactive networks among the critical genes involved in the Th1, Th2, Treg, Th17 and Th9 pathways, suggesting that these interactions were likely to modify an individual’s risk for the development of AR.

Although there is evidence that gene-gene interaction may contribute to the development and complexity of AR, presently only a few studies have detected an epistasis effect on several candidate genes[23–25]. However, other studies investigating associations between allergy and variants in the Th2-cell differentiation and signaling pathway provide good examples of gene–gene interactions[26–28]. For example, the analysis of genotyping data from a large population of German children revealed that when polymorphisms in IL4, IL13, IL4RA and STAT6 pathway genes were combined in a stepwise procedure, the risk for high serum IgE levels increased by 10.8-fold and the risk for the development of asthma increased by 16.8-fold, compared with the maximum effect of any individual SNP[26]. Furthermore, significant interactions were observed in a model with additive and dominant effects, for both pair and triplet combinations for asthma and for pairs of polymorphisms in IgE regulation[26]. Likewise, another multicenter study in German children has demonstrated significant interactions between IL4RA and IL13, and that individuals with the risk genotype for both genes were at almost five-times greater risk for the development of asthma compared with individuals with both non-risk genotypes[27]. One study in Chinese children with asthma, has demonstrated that there were significant interactions between IL13 and IL4RA for asthma and between IL13 and the thymus and activation-regulated chemokine (TARC) gene for increased plasma total IgE concentrations in this cohort[28]. In the present study, we specifically excluded subjects with comorbid asthma primarily to minimize the effect of asthma as a major confounding factor and focus specifically on candidate genes involved in susceptibility towards AR; particularly as we have recently demonstrated that some specific polymorphisms in key genes involved in Th17 pathways are potentially associated with comorbid asthma and AR[29]. Furthermore, it has been suggested that in addition to the "allergic disease genes," there are "phenotype-specific genes" or possibly certain combinations of susceptibility genes that contribute to the expression of asthma, allergic rhinitis, or atopic dermatitis[30]. Thus, under the premise of a limited study population, amixture of AR and asthma subjects was likely to demonstrate genetic associations which were not AR-specific, and thus emphasised the need to exclude subjects with co-morbid asthma from the present investigation.

Epistatic QTL-mapping studies in model organisms have detected many new interactions and it has been therefore concluded that epistasis may make a large contribution to the genetic regulation of complex traits[31]. Many studies indicate that epistatic gene action is common, and that additivity can be an emergent property of underlying genetic interaction networks[32]. Moreover, the epistatic interactions that have been detected may define previously uncharacterized, highly interconnected genetic networks that are enriched for biologically plausible gene ontology categories, metabolic and cellular pathways[32].

In the present trial study, epistasis analyses were performed for 91 tSNPs of 26 candidate genes selected from Treg, Th1, Th2, Th9 and Th17 pathways, which may contribute to the heterogeneous presentations of allergic diseases[33]. In this trial, 478 cases and 400 controls passed standard quality control measures and were assessed further, using a series of association analyses and the epistasis test, which showed 83 SNPs in 23 genes to be significantly interactive. STAT3, RORα and IL-26, all ofwhichareassociatedwith the Th 17 pathway, were the mostfrequentlyinteractive genes. Overall, 59interactions /SNP pairs demonstrated OR values higher than 2 or lower than 0.5 and 12 interactions/SNP pairs demonstrated OR values higher than 4 or lower than 0.25. Two interactions/SNP pairs, rs17405722 in STAT3 with rs6715106 in STAT4, and rs4913419 in IL-26 with rs2069870 in IL-9, demonstrated OR values lower than 0.1. In addition, the most significant interacting/SNP pair was rs1053005 in STAT3 with rs11574790 in IL-12 (OR = 0.24, *P* = 0.001). Although to our knowledge STAT3, either alone or in combination with another gene, has not been reported to play a significant role in the aetiology of AR, the findings from the present study suggest that STAT3 may significantly influence susceptibility to AR in Chinese subjects. Although simple SNP association analysis did notdetect significant associations among the Th2 related candidate genes, epistasis analysis did demonstrate that nine SNPs in these genes had significant interactive effects, thus supporting the notion that “each variant typically has modest effects in isolation, but synergizes effectively with other variants to magnify the impact on disease risk”[12].

The full modelresultsshowedthatrs2569254 in IL-12, rs1031508 in STAT4, and rs3741809 in IL-26 were likely to be recessive (*P* = 0.015, 0.024, 0.033, respectively);rs8193036 in IL17A likely to be allelic (*P* = 0.018);rs897200 in STAT4 likely to be genotypic (*P* = 0.026);and rs1898413 in RORα likely to be dominant (*P* = 0.039). Similarly, three significant SNPs; including rs8193036 in IL17A, rs2569254 in IL-12 and rs1898413 in RORα (OR = 0.78, 0.78, 1.30 and *P* = 0.019, 0.040, 0.044, respectively); weredetectedusinglogistic regression analysis. Overall, ourfindingsthatIL-12 and STAT4 are associated with the Th1 pathway, and IL17A, IL-26 and RORαareassociatedwith the Th17 pathway, are in agreementwith the findings from more recent studies, whichhaveindicatedthat Th1 and Th17 pathways play keyroles in AR[34,35]. Some studies, however, haveprovidedconflictiongdata for the role of IL-17A_ rs8193036 in allergy. While one study has reported a correlation between IL-17A SNP rs8193036 and paediatric asthma in Taiwanese children[36], another recent study has provided no such evidence of an association between IL-17A SNP rs8193036 with AR or AR accompanied with asthma in Chinese subjects[29]. Thus, from this perspective, it is tempting to speculate that IL-17 may possibly play a protective or an initiating role in AR susceptibility; depending on a variety of factors, including the pattern of sensitization, age, gender, environmental pollutants, etc; which need to be investigated in large well-defined study cohorts to elucidate the precise role of IL-17 in the genetics of AR.

Although the interaction or epistasis analyses in the present study are somewhat limited by the relatively small study groups, the preliminary findings from the study lend credibility to the biological plausibility of the underlying hypothesis and sheds new light on our understanding the mechanism of allergic disease. In particular, while the role of T-helper (Th1 and Th2) cells in the immunological mechanisms underlying allergic sensitization has been well documented, the contributions of T regulatory (Treg) cells and the proinflammatory Th17 cell lineage is less well understood. Similarly, the effect of gene-gene interactions; and particularly the role of specific interactions between variants in candidate genes which mediate signaling and transcriptional networks involving effector T-cell responsesare less well understood.Thus, in this context, our findings provide evidence that there are wide ranging interactions among the crucial genes involved in the effector T-cell pathways and that the Th17 pathway appears to be a key player in developing susceptibility to allergic rhinitis. Furthermore, these findings suggest that future studies involving genetic mechanisms underlying the pathogenesis of AR, as well as other allergic diseases, should focus on more complex models involving interactions between multiple candidate genes/polymorphisms, in order to take us closer to a truly comprehensive set of allergy susceptibility genes.However, the findings of the present study need to be confirmed in further studies involving a larger study cohortcomprising equal numbers of monosensitized and polysensitized subjects and assessing associations between a larger number of genes and potential mechanistic pathways involved in the etiology of AR.

## Supporting Information

S1 Table SNP-SNP interactions. (DOCX)
