# Supplementary material for: Association between the Interaction of Key Genes Involved in Effector T-Cell Pathways and Susceptibility to Developallergic Rhinitis: A Population-Based Case-Control Association Study
Source: PLoS One. 2015 Jul 21;10(7):e0131248. doi: 10.1371/journal.pone.0131248 (PMC4510440; doi:10.1371/journal.pone.0131248)
Supplement: S1 Table — (DOCX) [file pone.0131248.s001.docx]

**SUPPORTING INFORMATION**

**Table S1.** SNP-SNP interactions.

| Chr1 | SNP1 | Allele1 | Gene1 | Chr2 | SNP2 | Allele2 | Gene2 | OR | STAT | *P-value* |
| --- | --- | --- | --- | --- | --- | --- | --- | --- | --- | --- |
| 1 | rs3024495 | A | IL-10 | 10 | rs10752126 | G | GATA3 | 0.36 | 6.79 | 0.009 |
|  |  |  |  | 10 | rs406103 | A | GATA3 | 0.45 | 4.45 | 0.035 |
|  |  |  |  | 12 | rs3782555 | C | IL-26 | 2.33 | 4.24 | 0.040 |
|  |  |  |  | 15 | rs17237290 | G | RORα | 0.27 | 4.24 | 0.040 |
|  | rs3021094 | C |  | 5 | rs2243283 | C | IL-4 | 0.64 | 5.81 | 0.016 |
|  |  |  |  | 21 | rs8128785 | A | IFN-γ | 0.56 | 4.80 | 0.028 |
|  |  |  |  | 21 | rs2073362 | G | IFN-γ | 1.40 | 3.87 | 0.049 |
|  | rs3790622 | A |  | 4 | rs1598861 | C | NF-κB | 2.44 | 5.13 | 0.024 |
|  |  |  |  | 4 | rs2069762 | C | IL-2 | 0.49 | 7.73 | 0.005 |
|  | rs3024490 | C |  | 1 | rs1800893 | A | IL-10 | 0.52 | 4.31 | 0.038 |
|  |  |  |  | 2 | rs6715106 | G | STAT4 | 1.66 | 3.91 | 0.048 |
|  |  |  |  | 17 | rs17244587 | A | T-bet | 0.51 | 4.17 | 0.041 |
|  |  |  |  | 21 | rs2073362 | G | IFN-γ | 0.68 | 5.21 | 0.022 |
|  | rs1800893 | A |  | 4 | rs2069772 | G | IL-2 | 2.75 | 5.16 | 0.023 |
|  |  |  |  | 5 | rs2569253 | A | IL-12 | 0.59 | 4.38 | 0.036 |
|  |  |  |  | 10 | rs10752126 | G | GATA3 | 0.53 | 6.63 | 0.010 |
|  |  |  |  | 10 | rs406103 | A | GATA3 | 0.54 | 6.16 | 0.013 |
|  |  |  |  | 17 | rs3816769 | G | STAT3 | 1.85 | 5.27 | 0.022 |
|  |  |  |  | 21 | rs8128785 | A | IFN-γ | 3.41 | 3.89 | 0.049 |
| 2 | rs3771300 | C | STAT1 | 5 | rs2069868 | A | IL-9 | 0.65 | 4.93 | 0.026 |
|  |  |  |  | 5 | rs2569253 | A | IL-12 | 0.73 | 4.45 | 0.035 |
|  | rs7575823 | A |  | 2 | rs1031508 | A | STAT4 | 0.60 | 4.27 | 0.039 |
|  | rs2066804 | A |  | 5 | rs2069868 | A | IL-9 | 1.48 | 4.00 | 0.046 |
|  | rs2280235 | A |  | 5 | rs2069868 | A | IL-9 | 0.67 | 4.27 | 0.039 |
|  |  |  |  | 5 | rs2569253 | A | IL-12 | 0.71 | 5.95 | 0.015 |
|  |  |  |  | 5 | rs3212219 | A | IL-12 | 0.74 | 4.62 | 0.032 |
|  | rs34997637 | A |  | 2 | rs1031508 | A | STAT4 | 1.35 | 4.16 | 0.041 |
|  |  |  |  | 4 | rs1598861 | C | NF-κB | 0.68 | 4.65 | 0.031 |
|  |  |  |  | 21 | rs8128785 | A | IFN-γ | 1.79 | 4.09 | 0.043 |
|  | rs2030171 | G |  | 2 | rs1467199 | G | STAT1 | 0.68 | 5.55 | 0.019 |
|  |  |  |  | 2 | rs13017460 | A | STAT4 | 0.62 | 9.42 | 0.002 |
|  |  |  |  | 4 | rs1598861 | C | NF-κB | 1.51 | 5.20 | 0.023 |
|  |  |  |  | 10 | rs444929 | G | GATA3 | 0.57 | 3.88 | 0.049 |
|  | rs1467199 | G |  | 5 | rs31564 | A | IL-9 | 0.74 | 4.40 | 0.036 |
|  |  |  |  | 10 | rs369421 | G | GATA3 | 1.65 | 3.89 | 0.049 |
| 2 | rs6715106 | G | STAT4 | 7 | rs1800796 | C | IL-6 | 1.82 | 4.58 | 0.032 |
|  |  |  |  | 7 | rs13306433 | A | IL-6 | 4.44 | 5.55 | 0.018 |
|  |  |  |  | 15 | rs12594972 | G | RORα | 0.27 | 3.96 | 0.047 |
|  |  |  |  | 17 | rs17405722 | A | STAT3 | 0.10 | 6.79 | 0.009 |
|  | rs13017460 | A |  | 7 | rs1800796 | C | IL-6 | 1.62 | 9.08 | 0.003 |
|  |  |  |  | 7 | rs13306433 | A | IL-6 | 3.11 | 6.56 | 0.010 |
|  |  |  |  | 12 | rs324015 | A | STAT6 | 0.75 | 4.42 | 0.036 |
|  | rs11889341 | A |  | 6 | rs4711998 | G | IL17A | 0.65 | 6.36 | 0.012 |
|  | rs1551440 | G |  | 6 | rs3819025 | A | IL17A | 1.58 | 5.14 | 0.023 |
|  |  |  |  | 12 | rs7977932 | G | IL-31 | 0.39 | 9.33 | 0.002 |
|  |  |  |  | 17 | rs17405722 | A | STAT3 | 3.90 | 4.24 | 0.039 |
|  |  |  |  | 21 | rs11701402 | G | IFN-γ | 0.72 | 4.35 | 0.037 |
|  | rs7566274 | A |  | 4 | rs1598861 | C | NF-κB | 1.57 | 6.84 | 0.009 |
|  |  |  |  | 15 | rs12594972 | G | RORα | 0.35 | 5.72 | 0.017 |
|  |  |  |  | 15 | rs2162069 | G | RORα | 0.31 | 5.04 | 0.025 |
|  |  |  |  | 17 | rs3816769 | G | STAT3 | 0.70 | 5.37 | 0.020 |
|  | rs1031508 | A |  | 2 | rs897200 | G | STAT4 | 0.63 | 7.57 | 0.006 |
|  |  |  |  | 5 | rs2243248 | C | IL-4 | 2.27 | 6.04 | 0.014 |
|  |  |  |  | 5 | rs2243263 | G | IL-4 | 2.46 | 7.82 | 0.005 |
|  |  |  |  | 5 | rs2569254 | A | IL-12 | 1.46 | 4.18 | 0.041 |
|  |  |  |  | 17 | rs3816769 | G | STAT3 | 1.40 | 4.28 | 0.038 |
|  | rs897200 | G |  | 17 | rs3816769 | G | STAT3 | 0.74 | 4.31 | 0.038 |
| 4 | rs3774932 | G | NF-κB | 5 | rs2569254 | A | IL-12 | 1.48 | 4.60 | 0.032 |
|  |  |  |  | 6 | rs4711998 | G | IL17A | 1.64 | 8.08 | 0.004 |
|  |  |  |  | 6 | rs8193036 | A | IL17A | 1.43 | 5.20 | 0.023 |
|  |  |  |  | 12 | rs7977932 | G | IL-31 | 1.83 | 5.19 | 0.023 |
|  |  |  |  | 15 | rs12905435 | A | RORα | 1.83 | 5.67 | 0.017 |
|  |  |  |  | 15 | rs17237290 | G | RORα | 0.57 | 4.98 | 0.026 |
|  | rs1598861 | C |  | 12 | rs4913419 | A | IL-26 | 0.46 | 4.61 | 0.032 |
|  | rs4648037 | G |  | 5 | rs2069812 | G | IL-5 | 2.82 | 6.18 | 0.013 |
|  |  |  |  | 17 | rs11657388 | G | T-bet | 0.22 | 4.43 | 0.035 |
|  | rs4648110 | A |  | 4 | rs2069762 | C | IL-2 | 0.48 | 7.24 | 0.007 |
|  |  |  |  | 5 | rs2069744 | A | IL-13 | 0.37 | 5.48 | 0.019 |
|  |  |  |  | 6 | rs4711998 | G | IL17A | 2.08 | 6.03 | 0.014 |
| 4 | rs2069772 | G | IL-2 | 9 | rs1317230 | C | IL-33 | 1.60 | 4.16 | 0.041 |
|  |  |  |  | 9 | rs1332290 | C | IL-33 | 1.72 | 5.62 | 0.018 |
|  |  |  |  | 21 | rs8128785 | A | IFN-γ | 5.85 | 7.36 | 0.007 |
| 5 | rs2069812 | G | IL-5 | 12 | rs3741809 | G | IL-26 | 1.48 | 6.09 | 0.014 |
|  |  |  |  | 12 | rs2227501 | A | IL-22 | 0.45 | 9.48 | 0.002 |
|  |  |  |  | 12 | rs17224704 | A | IL-22 | 0.46 | 7.49 | 0.006 |
|  |  |  |  | 12 | rs2227481 | A | IL-22 | 0.67 | 3.96 | 0.047 |
| 5 | rs1881457 | C | IL-13 | 12 | rs3782555 | C | IL-26 | 0.66 | 5.59 | 0.018 |
|  | rs2069744 | A |  | 5 | rs2243283 | C | IL-4 | 4.55 | 8.19 | 0.004 |
|  |  |  |  | 6 | rs3819025 | A | IL17A | 0.52 | 4.15 | 0.042 |
|  |  |  |  | 12 | rs3741809 | G | IL-26 | 1.68 | 3.90 | 0.048 |
| 5 | rs2243248 | C | IL-4 | 7 | rs1800796 | C | IL-6 | 0.32 | 9.65 | 0.002 |
|  | rs2243263 | G |  |  |  |  |  | 0.41 | 6.60 | 0.010 |
| 5 | rs31564 | A | IL-9 | 5 | rs2069870 | G | IL-9 | 2.68 | 4.20 | 0.040 |
|  |  |  |  | 5 | rs3212219 | A | IL-12 | 1.40 | 5.61 | 0.018 |
|  |  |  |  | 9 | rs1317230 | C | IL-33 | 0.72 | 5.63 | 0.018 |
|  |  |  |  | 9 | rs1332290 | C | IL-33 | 0.69 | 7.16 | 0.007 |
|  |  |  |  | 12 | rs167769 | A | STAT6 | 0.62 | 8.52 | 0.004 |
|  |  |  |  | 12 | rs11177102 | A | IL-26 | 0.76 | 4.04 | 0.044 |
|  |  |  |  | 15 | rs12905435 | A | RORα | 0.58 | 4.83 | 0.028 |
|  |  |  |  | 15 | rs1898413 | A | RORα | 0.67 | 4.51 | 0.034 |
|  | rs2069870 | G |  | 7 | rs1800796 | C | IL-6 | 2.77 | 4.76 | 0.029 |
|  |  |  |  | 10 | rs10752126 | G | GATA3 | 2.41 | 4.69 | 0.030 |
|  |  |  |  | 12 | rs4913419 | A | IL-26 | 0.08 | 4.98 | 0.026 |
|  | rs2069868 | A |  | 6 | rs4711998 | G | IL17A | 0.58 | 6.24 | 0.012 |
|  |  |  |  | 9 | rs1317230 | C | IL-33 | 0.63 | 5.15 | 0.023 |
|  |  |  |  | 10 | rs444929 | G | GATA3 | 0.37 | 5.19 | 0.023 |
|  |  |  |  | 15 | rs17237290 | G | RORα | 0.51 | 4.58 | 0.032 |
| 5 | rs11574790 | A | IL-12 | 12 | rs1182844 | T | IL-22 | 0.43 | 5.29 | 0.021 |
|  |  |  |  | 12 | rs1179251 | G | IL-22 | 0.33 | 6.64 | 0.010 |
|  |  |  |  | 17 | rs1053005 | G | STAT3 | 0.24 | 10.72 | 0.001 |
|  |  |  |  | 21 | rs1131964 | A | IFN-γ | 2.40 | 4.49 | 0.034 |
|  | rs2569253 | A |  | 12 | rs11177102 | A | IL-26 | 0.75 | 3.96 | 0.047 |
|  |  |  |  | 12 | rs3782555 | C | IL-26 | 1.54 | 8.45 | 0.004 |
|  |  |  |  | 15 | rs11635975 | G | RORα | 0.69 | 5.12 | 0.024 |
|  | rs2569254 | A |  | 15 | rs11635975 | G | RORα | 1.67 | 5.61 | 0.018 |
|  | rs3212219 | A |  | 12 | rs11177102 | A | IL-26 | 0.73 | 4.56 | 0.033 |
|  |  |  |  | 12 | rs3782555 | C | IL-26 | 1.44 | 6.03 | 0.014 |
|  |  |  |  | 15 | rs11635975 | G | RORα | 0.67 | 5.45 | 0.020 |
| 6 | rs4711998 | G | IL17A | 21 | rs2073362 | G | IFN-γ | 0.54 | 9.63 | 0.002 |
|  | rs8193036 | A |  | 7 | rs1800796 | C | IL-6 | 0.68 | 5.23 | 0.022 |
|  |  |  |  | 14 | rs10137082 | A | IL-25 | 1.47 | 3.89 | 0.049 |
|  | rs3819024 | G |  | 7 | rs13306433 | A | IL-6 | 2.96 | 5.22 | 0.022 |
|  |  |  |  | 14 | rs10137082 | A | IL-25 | 0.69 | 4.16 | 0.041 |
|  | rs2275913 | A |  | 10 | rs10752126 | G | GATA3 | 1.36 | 4.74 | 0.029 |
|  |  |  |  | 10 | rs406103 | A | GATA3 | 1.37 | 4.35 | 0.037 |
|  |  |  |  | 17 | rs1053005 | G | STAT3 | 1.38 | 3.90 | 0.048 |
|  | rs3819025 | A |  | 10 | rs10752126 | G | GATA3 | 0.58 | 7.28 | 0.007 |
|  |  |  |  | 10 | rs406103 | A | GATA3 | 0.62 | 5.46 | 0.019 |
|  |  |  |  | 15 | rs2162069 | G | RORα | 3.51 | 4.22 | 0.040 |
|  | rs3748067 | A |  | 17 | rs1053005 | G | STAT3 | 0.66 | 3.97 | 0.046 |
| 7 | rs1800796 | C | IL-6 | 15 | rs17237290 | G | RORα | 1.69 | 4.02 | 0.045 |
|  |  |  |  | 17 | rs17405722 | A | STAT3 | 0.30 | 4.91 | 0.027 |
|  |  |  |  | 17 | rs17244587 | A | T-bet | 0.48 | 5.14 | 0.023 |
| 9 | rs1317230 | C | IL-33 | 12 | rs4913419 | A | IL-26 | 2.01 | 4.86 | 0.028 |
|  |  |  |  | 17 | rs3816769 | G | STAT3 | 1.44 | 5.81 | 0.016 |
|  | rs1332290 | C |  | 17 | rs3816769 | G | STAT3 | 1.40 | 4.81 | 0.028 |
| 10 | rs369421 | G | GATA3 | 10 | rs406103 | A | GATA3 | 0.49 | 4.83 | 0.028 |
|  |  |  |  | 12 | rs3782555 | C | IL-26 | 0.53 | 5.91 | 0.015 |
|  |  |  |  | 12 | rs1182844 | T | IL-22 | 1.82 | 6.29 | 0.012 |
|  |  |  |  | 12 | rs1179251 | G | IL-22 | 1.93 | 6.41 | 0.011 |
|  |  |  |  | 15 | rs17237290 | G | RORα | 3.99 | 8.53 | 0.003 |
|  |  |  |  | 15 | rs2289163 | C | RORα | 2.97 | 5.85 | 0.016 |
| 12 | rs324015 | A | STAT6 | 15 | rs2162069 | G | RORα | 0.24 | 6.10 | 0.013 |
| 12 | rs4913419 | A | IL-26 | 12 | rs1182844 | T | IL-22 | 1.97 | 4.36 | 0.037 |
|  | rs3741809 | G |  | 17 | rs17405722 | A | STAT3 | 4.27 | 6.29 | 0.012 |
|  | rs11177102 | A |  | 17 | rs1053005 | G | STAT3 | 1.39 | 4.29 | 0.038 |
|  |  |  |  | 17 | rs17405722 | A | STAT3 | 0.17 | 7.18 | 0.007 |
| 12 | rs2227501 | A | IL-22 | 17 | rs1053005 | G | STAT3 | 1.71 | 4.72 | 0.030 |
|  |  |  |  | 17 | rs3816769 | G | STAT3 | 1.63 | 4.93 | 0.026 |
|  | rs2046068 | C |  | 17 | rs17405722 | A | STAT3 | 0.33 | 4.38 | 0.036 |
|  | rs17224704 | A |  | 17 | rs3816769 | G | STAT3 | 1.64 | 4.24 | 0.040 |
|  | rs2227481 | A |  | 17 | rs17405722 | A | STAT3 | 0.29 | 5.15 | 0.023 |
| 12 | rs7977932 | G | IL-31 | 17 | rs3816769 | G | STAT3 | 1.67 | 3.94 | 0.047 |
| 14 | rs10137082 | A | IL-25 | 15 | rs12905435 | A | RORα | 2.07 | 3.92 | 0.048 |
|  | rs10135798 | G |  | 15 | rs11635975 | G | RORα | 3.68 | 4.07 | 0.044 |
|  |  |  |  | 15 | rs2289163 | C | RORα | 0.20 | 4.14 | 0.042 |
| 15 | rs1898413 | G | RORα | 15 | rs2162069 | G | RORα | 0.22 | 3.92 | 0.048 |
|  | rs11635975 | A |  | 15 | rs2289163 | G | RORα | 0.54 | 4.38 | 0.036 |
|  | rs12594972 | G |  | 21 | rs11701402 | C | IFN-γ | 2.70 | 4.35 | 0.037 |
| 17 | rs1053005 | G | STAT3 | 17 | rs17244587 | A | T-bet | 0.36 | 8.89 | 0.003 |
|  | rs3816769 | G |  |  |  |  |  | 0.45 | 6.40 | 0.011 |

Note: SNP, single nucleotide polymorphisms; Chr1, chromosome of first SNP; SNP1, Identifier for first SNP; Allele1, risk allele of first SNP; Chr2, chromosome of second SNP; SNP1, Identifier for second SNP; Allele2, risk allele of second SNP; OR, odds ratio; STAT, Chi-square statistic.

^JAI GANPATI BAPA JAI CHAMUNDA MATA^
